# Tactile Detection in Fibromyalgia: A Systematic Review and a Meta-Analysis

**DOI:** 10.3389/fpain.2021.740897

**Published:** 2021-12-24

**Authors:** Tania Augière, Audrey Desjardins, Emmanuelle Paquette Raynard, Clémentine Brun, Anne Marie Pinard, Martin Simoneau, Catherine Mercier

**Affiliations:** ^1^Center for Interdisciplinary Research in Rehabilitation and Social Integration, Quebec City, QC, Canada; ^2^Department of Rehabilitation, Faculty of Medicine, Laval University, Quebec City, QC, Canada; ^3^Laval University Library, Laval University, Quebec City, QC, Canada; ^4^Department of Anesthesiology and Intensive Care, Faculty of Medicine, Laval University, Quebec City, QC, Canada; ^5^Department of Kinesiology, Faculty of Medicine, Laval University, Quebec City, QC, Canada

**Keywords:** chronic pain, somatosensory, quantitative sensory testing, touch, integration

## Abstract

Fibromyalgia is a chronic pain syndrome characterized by sensorimotor deficits and distortions of body representation, that could both be caused by alterations in sensory processing. Several studies suggest a hypersensitivity to various sensory stimulations in fibromyalgia but results on detection of both noxious and non-noxious tactile stimulation, which are particularly relevant for body representation and motor control, remain conflicting. Therefore, the aim of this study is to systematically review and quantify the detection thresholds to noxious and non-noxious tactile stimuli in individuals with fibromyalgia compared to pain-free controls. A systematic review and a meta-analysis were performed in the MEDLINE, EMBASE, CINAHL, Cochrane, PsycInfo and Web of Science databases using keywords related to fibromyalgia, tactile pain detection threshold, tactile detection threshold and quantitative sensory testing. Nineteen studies were included in the review, with 12 in the meta-analysis. Despite the heterogeneity of the results, the data from both the review and from the meta-analysis suggest a trend toward hyperalgesia and no difference of sensitivity to non-noxious tactile stimuli in participants with fibromyalgia compared to healthy controls. This contradicts the hypothesis of a general increase in responsiveness of the central nervous system to noxious and non-noxious stimulations in fibromyalgia. This study shows no alteration of the sensitivity to non-noxious tactile stimulation in fibromyalgia, suggesting that an altered unimodal processing is not sufficient to explain symptoms such as sensorimotor impairments and body representation distortions. Future research should investigate whether alterations in multisensory integration could contribute to these symptoms.

## Introduction

Fibromyalgia is a chronic widespread pain syndrome that affects 2% of the world population (1, 2) and is characterized by various symptoms including sensorimotor deficits (3–5) and distortions of body representation (6–10). These symptoms could partly stem from an altered processing of sensory information. Intact unimodal processing of sensory information is essential for subsequent integration of these signals among other information (i.e. multimodal integration), which is at the core of body representation (11, 12) and motor control (13–15).

An extensive literature on unimodal processing of sensory information suggests that persons with fibromyalgia are hypersensitive to auditory stimulations (16–20), olfactory stimulations (21), and somatosensory stimulations, such as thermal (22–32), pressure (22, 24, 25, 27, 28, 30, 32–36) and electrical stimulations (22, 33, 37). In fact, the presence of body areas hypersensitive to pressure, called tender points, is one of the most common symptoms of fibromyalgia (38). This hyperalgesia (i.e., hypersensitivity to noxious stimuli) is often accompanied by allodynia [i.e., a painful response to non-noxious stimuli such as light touch or warmth (39)] and is not restricted to tender points (40, 41). Several studies show an altered perception of both noxious and non-noxious stimulations in fibromyalgia (24, 28, 30, 42). Using thermal stimulation, Kosek et al. (41) demonstrated an increased sensitivity to noxious and non-noxious stimulations on sites of maximal pain and a decreased sensitivity to both types of stimulation on sites of minimal pain. However, other authors reported contradictory findings regarding the altered perception of non-noxious stimuli (22, 31, 43–45) or its direction (42). These somatosensory alterations are measured with Quantitative Sensory Testing (QST), a standardized method used to assess sensory system functioning (46). The test procedure enables to obtain and to compare the detection thresholds to various stimuli (tactile, thermal, pressure etc.) between groups of individuals. A recent systematic review (47) examined the perception of thermal stimulation in fibromyalgia and showed that most studies using QST reported hyperalgesia to cold (82% of the studies) and heat stimulations (77% of the studies) in fibromyalgia but no alterations in the detection of non-noxious cold stimulations (70.6%). However, results on the alterations of perception of noxious and non-noxious stimuli for other sensory modalities in fibromyalgia remain conflicting.

Processing of tactile stimulation has been highlighted as particularly relevant for body representation (48) and motor control (49), two aspects that have been shown to be altered in individuals with fibromyalgia. Therefore, the aim of the present study is to systematically review and quantify the detection thresholds to noxious and non-noxious tactile stimuli in individuals with fibromyalgia compared to pain-free controls. According to the symptomatology of fibromyalgia, it is hypothesized that individuals with fibromyalgia would exhibit hypersensitivity to both noxious (i.e., hyperalgesia) and non-noxious stimuli (hyperesthesia).

## Methods

This systematic review was conducted in accordance with the PRISMA statement (50).

The protocol of the review was registered in PROSPERO (www.crd.york.ac.uk/prospero), an international database for the prospective registration of systematic reviews on the 08/27/20 (registration number: CRD42020198167).

### Search Strategy

A systematic search was conducted on the 06/18/20 by a professional librarian in the electronic databases MEDLINE, EMBASE, CINAHL, Cochrane, PsycInfo and Web of Science (see Supplementary Material). The search was restricted to studies in English and in French, with no publication date limit. Keywords used for the search included “Fibromyalgia,” “Quantitative sensory testing,” “Tactile detection threshold,” and “Tactile pain detection threshold.” The strategy was personalized for each database. An additional manual search was performed in the reference lists of the included articles. Search results were exported to Covidence (www.covidence.org), a systematic review management website, for automatic duplicates removal.

### Inclusion and Exclusion Criteria

Only peer-reviewed papers were included in the systematic review. To be included, studies had to include (1) a fibromyalgia group and a pain-free group; and (2) a measure of tactile detection thresholds (TDT) or tactile pain detection thresholds (TPT). Comorbidities could be present in the fibromyalgia group and there was no age restriction for either participant groups. Animal studies and studies written neither in English nor in French were excluded.

### Articles Selection

The selection process followed 2 steps. First, two reviewers (T.A. and A.D.) independently screened the titles and abstracts of the articles according to the inclusion and exclusion criteria. If there was any uncertainty regarding the eligibility of a study, the paper was kept for the second step. Then, the two reviewers independently reviewed the full text of the selected articles, still according to the inclusion and exclusion criteria. For each step, an agreement between the two reviewers had to occur to include the article. If a disagreement arose, a discussion between the two reviewers occurred to reach a consensus, or the judgement of a third author (C.B. or C.M.) was sought if needed.

### Quality Assessment

The quality of the included studies was independently assessed by the two reviewers according to the Standard quality assessment criteria for evaluating primary research papers from a variety of fields (51). This quality assessment tool includes 14 criteria, each evaluated on a 2-point scale (2 – element sufficiently described, and no added bias introduced in the results; 1 – element not sufficiently described but no added bias introduced in the results; 0 – element not mentioned or added bias in the results). The quality score of each study was expressed as a percentage and interpreted with the following scale (52): 90% and more is a very high quality, 80–89% is a high quality, 70–79% is a moderate quality, 60–69% is a low quality, 59% and less is a very low quality. Because three of the 14 criteria related to interventional studies, only 11 criteria were used for quality assessment.

The Gwet's coefficient was calculated for each criterion of the quality scale to evaluate the inter-rater agreement (53). The agreement was interpreted as poor (inferior to 0.0), slight (0.0 to 0.20), fair (0.21 to 0.40), moderate (0.41 to 0.60), substantial (0.61 to 0.80), or almost perfect [0.81 to 1.00 (53)]. Any discrepancy between the evaluation of the two reviewers was resolved by a consensual decision, or the judgement of a third author was sought if needed.

### Data Extraction

The following variables were extracted from the included articles:

1) sample data (number of participants in each group, age, sex, mean pain intensity of the participants with fibromyalgia, whether medication was stopped prior to participation);
2) methodological characteristics (threshold type, tool used, threshold assessment method, threshold definition, stimulated sites and whether they were painful in the fibromyalgia group, order of the presentation if several modalities of stimulation were tested in the same study, statistical tests performed);
3) the results (means and standard deviations of the thresholds for each group according to the stimulated site, *t*- or *F*-value, *p*-value).

### Meta-Analysis

In order to quantify the threshold difference between the fibromyalgia and the control group, Cohen's d, a quantification of effect size (54), was calculated using the website EffectSizeCalculator (www.campbellcollaboration.org/escalc/html/EffectSizeCalculator-SMD1.php). If data were missing to calculate Cohen's d, an email was sent to the corresponding author to obtain the required data. In our study, a positive d reflected a hyposensitivity (hypoesthesia or hypoalgesia, for non-noxious and noxious stimuli, respectively), to tactile stimuli in fibromyalgia and a negative d was associated to a hypersensitivity (hyperesthesia or hyperalgesia) in fibromyalgia.

To synthesize the effect sizes of the included studies, a global effect size D was calculated with an univariate random-effects model (55) for each threshold (TDT and TPT) using R Studio (RStudio, Inc., Boston, MA). The random-effects model accounts for the variability between the studies included. D was calculated using one effect d per study because the conditions of application of the effect size prohibits the use of dependent samples. Hence, the “distal upper limb area” (hand, wrist, forearm) was chosen as a common stimulation area. This area was selected because (1) it was the most frequently stimulated area across studies and therefore allowed for a larger number of studies to be included in the meta-analysis and (2) it is not a tender point in fibromyalgia and thus it would indicate whether somatosensory alterations in fibromyalgia were generalized to non-tender sites. The studies excluded from the meta-analysis were synthesized in the qualitative analysis to assess the risk of bias due to missing results in the meta-analysis.

Heterogeneity was assessed with Cochran's Q (56).

## Results

### Selection Process

**Figure 1:**
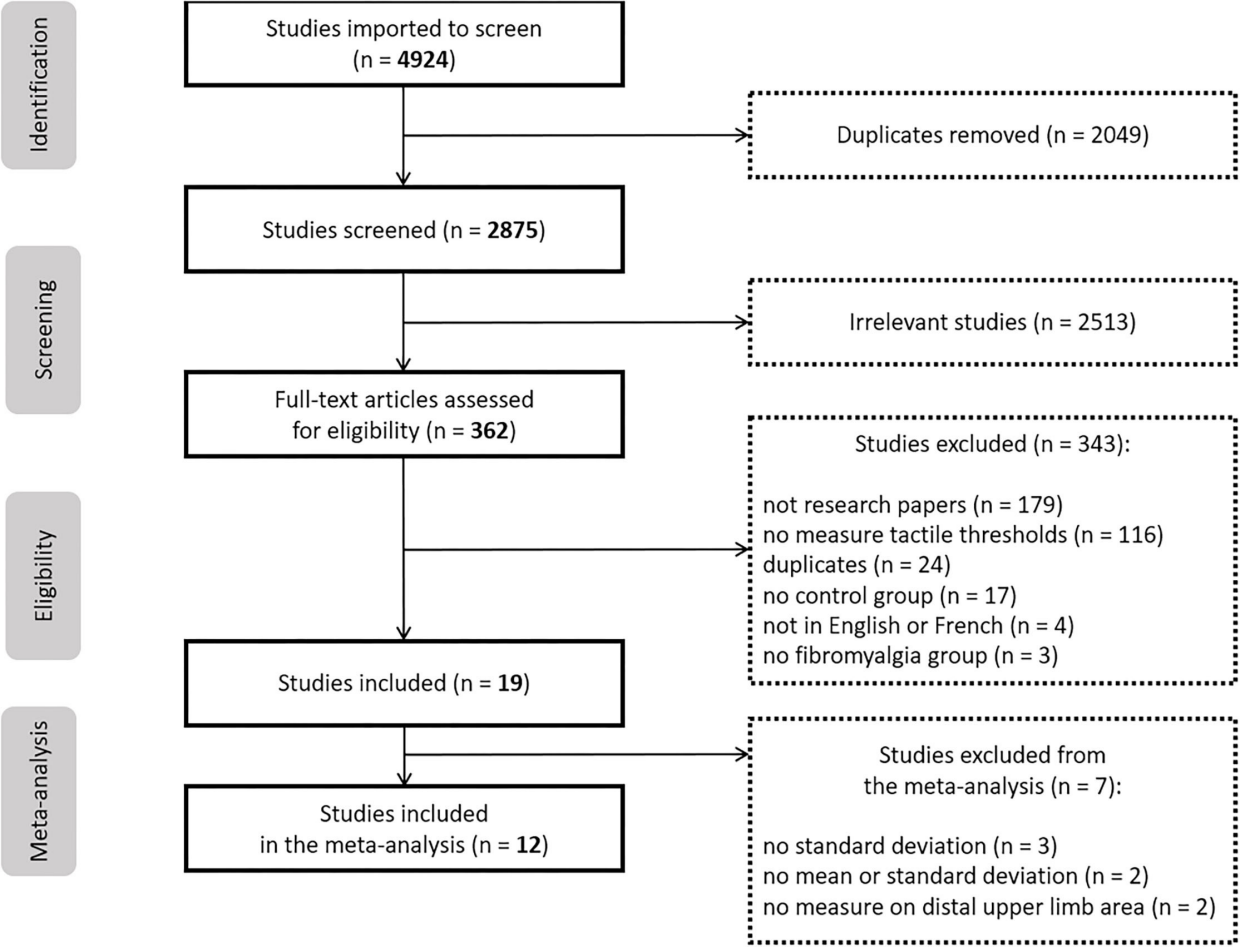
PRISMA flowchart.

The electronic literature search yielded 4,924 articles, among which 2,049 duplicates were removed by Covidence. The screening of titles and abstracts led to the exclusion of 2,513 additional articles. The full-text analysis led to the exclusion of 343 supplementary articles because there were not scientific papers (*n* = 179), they did not measure tactile thresholds (*n* = 116), they were duplicates (*n* = 24), they did not include a pain-free control group (*n* = 17), they were not written in English nor in French (*n* = 4) or they did not include a fibromyalgia group (*n* = 3). The selection process is reported in the PRISMA flow-chart (Figure 1). In total, 19 studies were included in the systematic review. The extraction table is available in the Supplementary Material.

Of these 19 studies, 14 included TDT assessments and 14 included TPT assessments. Twelve articles were included in the meta-analysis, for a total of eight studies with TDT assessment and nine studies with TPT assessment (some studies assessing both types of thresholds). The remaining seven studies were excluded from the meta-analysis because: 1- the standard deviation of the threshold was missing (*n* = 3); 2- the threshold was only expressed in z-score (*n* = 2); 3- the distal upper limb area was not studied (*n* = 2). The corresponding authors of these studies were contacted to obtain the missing data, but they either did not respond (*n* = 5) or could not provide the missing information (*n* = 2).

### Quality Assessment

According to Gwet's coefficient, the mean inter-rater agreement across all criteria was almost perfect (0.81 ± 0.15). Among the 11 criteria, the coefficient ranged from 0.57 to 1.

After consensus between the two reviewers, the mean quality score was high (80.9 ± 7.7%) and ranged from 65.9% (low) to 90.9% (very high) across all the studies.

### Study Characteristics

#### Demographic Characteristics

In total 690 individuals with fibromyalgia and 643 pain-free controls were included. All participants were adults (mean age of 45.7 ± 6.2). 95.7% of participants with fibromyalgia and 86.6% of pain-free controls were women [one study did not indicate the proportion of men and women (57)]. Fourteen studies out of the 19 reported a mean pain intensity rating, either at the moment of the participation [*n* = 10; (24, 27, 31, 34, 36, 37, 57–60)] or within the last month prior [*n* = 4; (30, 33, 35, 61)]. In nine studies, including five in the meta-analysis (32, 57, 59, 61, 62), individuals with fibromyalgia were instructed to stop taking their medication prior to their participation (27, 30, 32, 33, 57, 59, 61–63).

For the meta-analysis, 474 individuals with fibromyalgia and 461 pain-free controls were included. The mean age was 45.4 ± 7.4 years, 94.3% of participants with fibromyalgia and around 85.4% of pain-free controls were women.

#### Assessment Methods

The TDT was assessed with Von Frey filaments in all studies except one (60) in which unspecified nylon filaments were used. For the TPT, five studies involved pinprick stimulators (27, 28, 30, 32, 35, 36, 61), five involved Von Frey filaments (34, 37, 57, 62, 63), one involved unspecified nylon filaments (60) and one involved disposable needles (59).

Out of the 19 studies, 15 used the method of limits (24, 27, 28, 30, 32, 34–37, 41, 57, 60–63), one the staircase method (58) and three did not report the method used (31, 33, 59). In the method of limits, the stimulus intensity increases or decreases continuously, and the participants have to say when they detect or stop detecting the stimulation (46), whereas in the staircase method, the stimulus intensity increases or decreases according to whether the participant detects the stimulus (64).

The most frequently stimulated site was the hand [13 studies (24, 27, 28, 30, 31, 34, 35, 57–62)], then the back [seven studies (27, 28, 30, 33, 37, 60, 61)], the foot (30, 35, 36), the face (28, 59, 60) and the forearm (32, 37, 63); three studies each, and the elbow (58), the tibia (59), and the sternum (24) (one study each).

### Thresholds Comparisons

#### Comparison of Tactile Detection Thresholds

Three studies reported a hypoesthesia in persons with fibromyalgia compared to controls (34, 36, 60), whereas eight studies reported no difference between the groups (27, 30, 31, 33, 35, 58, 59, 61). The remaining three studies reported either no significant difference or a hypoesthesia, depending on the site stimulated (24, 28, 41). As shown in Table 1, the studies reporting a significant difference between the groups were not of better or lower quality than the studies reporting no difference. Moreover, no substantial differences were identified across these studies in terms of methodology, sample sizes, or mean pain ratings.

**Table 1:** Studies included in the review with measures of TDT.

						**Results**			
**References**	**FM:HC**	**Female/** **MALE**	**Mean age ± SD (range)**	**Pain intensity ± SD (range)**	**Medication stopped?**	**FM < HC**	**FM > HC**	**NS**	**Quality**
Kaziyama et al. (34)	32:31:00	32/0	45.9 ± 8.5	Subgroup 1: 22.7 ± 7.5/100 Subgroup 2: 28.3 ± 3.9/100	?		Hand dorsum, thenar		86.4%
de Siqueira et al. (59)	8:41	8/0	47.0 ± 1.2	9.0 ± 1.7/10	Yes			Ophtalmic branch, maxillar branch, mandibular branch, hand dorsum, tibia (grouped together)	65.9%
Hilgenberg-Sydney et al. (60)	20:20	20/0	50.0 ± 6.8	43.6 ± 24.9/100	?		Masseter, thenar, cervical		84.1%
Gerhardt et al. (61)	90:40	80/10	55.1 ± 9.3	6/10	Yes			Lumbar, *hand dorsum*	88.6%
Palmer et al. (24)	36:37	28/8	51.0 ± 9.85	5.7 ± 1.3/10	No		Index of left (for most participants) arm	Sternum, index of right (for most participants) arm	90.9%
Hurtig et al. (31)	29:21	29/0	46 (30; 68)	47.3/100 (12.5; 75)	?			Hand dorsum	81.8%
Lim et al. (33)	19:21	19/0	44.9 ± 8.3	57.2 ± 20.1/100	Yes			Trapezius, hand dorsum	84.1%
Klauenberg et al. (35)	35:25	30/5	48.0 ± 9.0	5 ± 2/10	No			Palm hand, dorsum foot	90.9%
Blumenstiel et al. (27)	21:20	21/0	50.6 ± 9.5	6.8 ± 1.8/10	Yes			Back, *hand dorsum*	84.1%
Evdokimov et al. (36)	117:178	117/0	52.0 (22; 75)	5/10 (0; 9)	No		Dorsum foot		84.1%
Kosek et al. (41)	10:10	10/0	42.7 (25, 60)	?	No		Site contralateral to site of maximum pain	Site of maximum pain area, *site of minimum pain area*, site contralateral to site of minimum pain	72.7%
Martinez-Jauand et al. (58)	113:65	113/0	51.1 ± 8.8	Subgroup 1: 7.6 ± 1.7/10 subgroup 2: 6.9 ± 1.7/10	no			Ventral wrist, elbow, index	81.8%
Pfau et al. (28)	14:18	13/1	50.6 ± 5.1	?	No		Cheek, trapezius	Hand dorsum	81.8%
Tampin et al. (30)	22:31	20/2	46.1 ± 11.5	7.3 ± 1.2/10	Yes			Maximum pain site, *dorsum foot*, dorsum hand, thenar	86.4%

*FM, participants with fibromyalgia; HC, healthy controls; SD, standard deviation; NS, non-significant*.

*Question marks indicate non reported data. The studies in the gray rectangle at the top were also included in the meta-analysis. Underlined sites are painful sites in participants with fibromyalgia whereas sites in italics are not painful. The first set of columns describes the participants, the second set of columns describes the results of the comparisons between the two groups, and the last column reports the quality score*.

**Figure 2:**
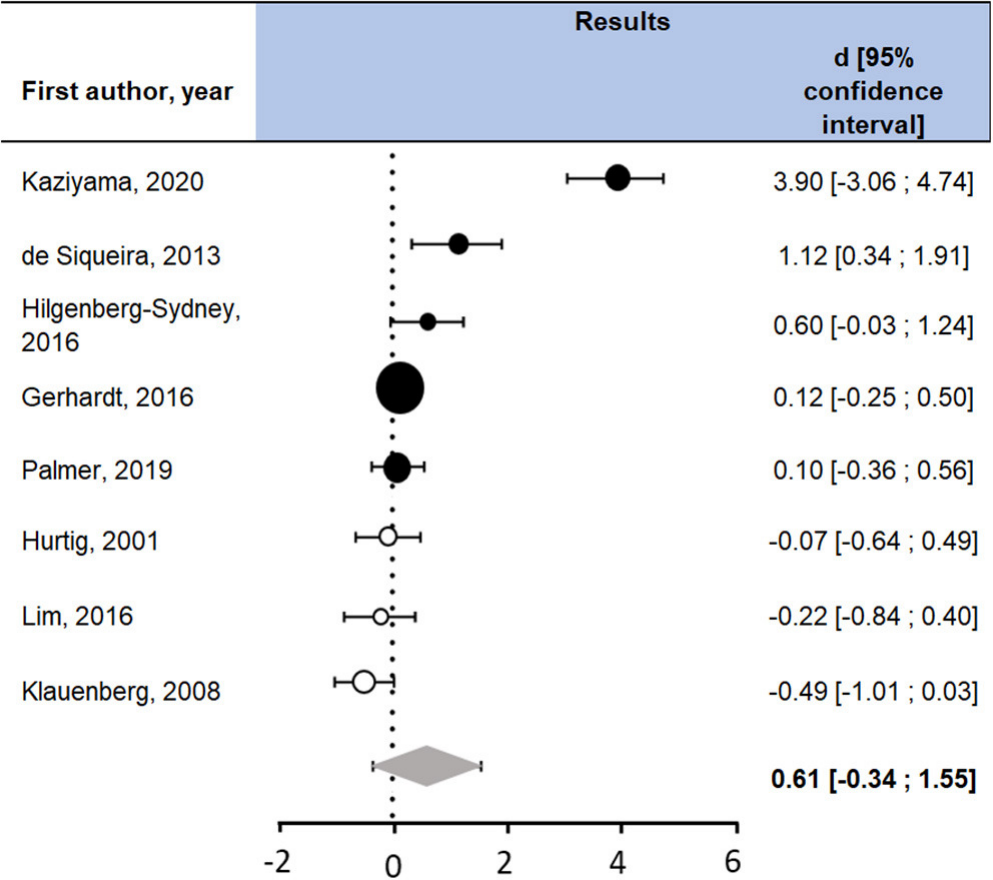
Studies included in the meta-analysis with measures of TDT. FM, participants with fibromyalgia; HC, healthy controls; SD, standard deviation. Question marks indicate non-reported data. A positive d (in black) can be interpreted as a hypoalgesia in participants with fibromyalgia compared to healthy controls, whereas a negative d (in white) means there is a hyperalgesia in participants with fibromyalgia compared to healthy controls. The summarized Cohen's d is represented by the gray diamond. Confidence intervals containing zero means the d is not statistically significant.

The eight studies included in the meta-analysis yielded a non-significant summarized effect size D of 0.61 (Figure 2). Cochran's Q revealed a significant heterogeneity in the studies' results [Q (df = 7) = 90.26, *p* < 0.0001]. The study with the extreme result (34) was of high quality (score of 86%). No notable difference was found between this study and the rest of the studies included in the meta-analysis.

Given the heterogeneity of the results, it is difficult to draw a conclusion regarding the difference of perception of non-noxious tactile stimulation between individuals with fibromyalgia and controls.

#### Comparison of Tactile Pain Detection Thresholds

Six studies reported a hyperalgesia in participants with fibromyalgia compared to controls (27, 32, 34, 57, 60, 62) whereas two reported a hypoalgesia (36, 59) and four reported no significant difference between the groups (30, 35, 61, 63). In two studies, either no significant difference or an hyperalgesia was observed, depending on the stimulated site (28, 37). As shown in Table 2, no notable differences in quality scores, methodology, sample sizes, or mean pain ratings were found between the studies reporting different results.

**Table 2:** Studies included in the review with measures of TPT.

						**Results**			
**References**	**FM:HC**	**Female/male**	**Mean age ± SD (range)**	**Pain intensity ± SD (range)**	**Medication stopped?**	**FM < HC**	**FM > HC**	**NS**	**Quality**
de Siqueira et al. (59)	8:41	8/0	47.0 ± 1.2	9.0 ± 1.7/10	Yes		Ophtalmic branch, maxillar branch, mandibular branch, hand dorsum, tibia (grouped together)		65.9%
Gerhardt et al. (61)	90:40	80/10	55.1 ± 9.3	6/10	Yes			Lumbar, *hand dorsum*	88.6%
Klauenberg et al. (35)	35:25	30/5	48.0 ± 9.0	5 ± 2/10	No			Palm hand, dorsum foot	90.9%
van Laarhoven et al. (37)	15:19	15/0	44.5 ± 7.9	5.4 ± 2.0/10	No	*Trapezius*		Forearm	77.3%
Kaziyama et al. (34)	32:31	32/0	45.9 ± 8.5	Subgroup 1: 22.7 ± 7.5/100 Subgroup 2: 28.3 ± 3.9/100	?	Hand dorsum, thenar			86.4%
Carli et al. (57)	60:22	58/2	44.2 ± 9.8	70.7 ± 4.7/100	Yes	Index			75.0%
Crettaz et al. (32)	13:10	13/0	49.9 ± 10.6	?	Yes	Forearm			68.2%
Hilgenberg-Sydney et al. (60)	20:20	20/0	50.0 ± 6.8	43.6 ± 24.9/100	?	Masseter, thenar, cervical			84.1%
Eken et al. (62)	19:17	17/2	37.7 ± 5.8	?	Yes	Thumb			79.5%
Blumenstiel et al. (27)	21:20	21/0	50.6 ± 9.5	6.8 ± 1.8/10	Yes	Back, *hand dorsum*			84.1%
Burgmer et al. (63)	17:17	17/0	52.6 ± 8.0	?	Yes			Forearm	72.7%
Evdokimov et al. (36)	117:178	117/0	52.0 (0; 9)	5/10 (0; 9)	No		Dorsum foot		84.1%
Pfau et al. (28)	14:18	13/1	50.6 ± 5.1	?	No	Trapezius		Hand dorsum, cheek	81.8%
Tampin et al. (30)	22:31	20/2	46.1 ± 11.5	7.3 ± 1.2/10	Yes			Maximum pain site, *dorsum foot*, dorsum hand, thenar	86.4%

*FM, participants with fibromyalgia; HC, healthy controls; SD, standard deviation; NS, non-significant. Question marks indicate non reported data. The studies in the gray rectangle at the top were also included in the meta-analysis. Underlined sites are painful sites in participants with fibromyalgia whereas sites in italics are not painful. The first set of columns describes the participants, the second set of columns describes the results of the comparisons between the two groups, and the last column reports the quality score*.

**Figure 3:**
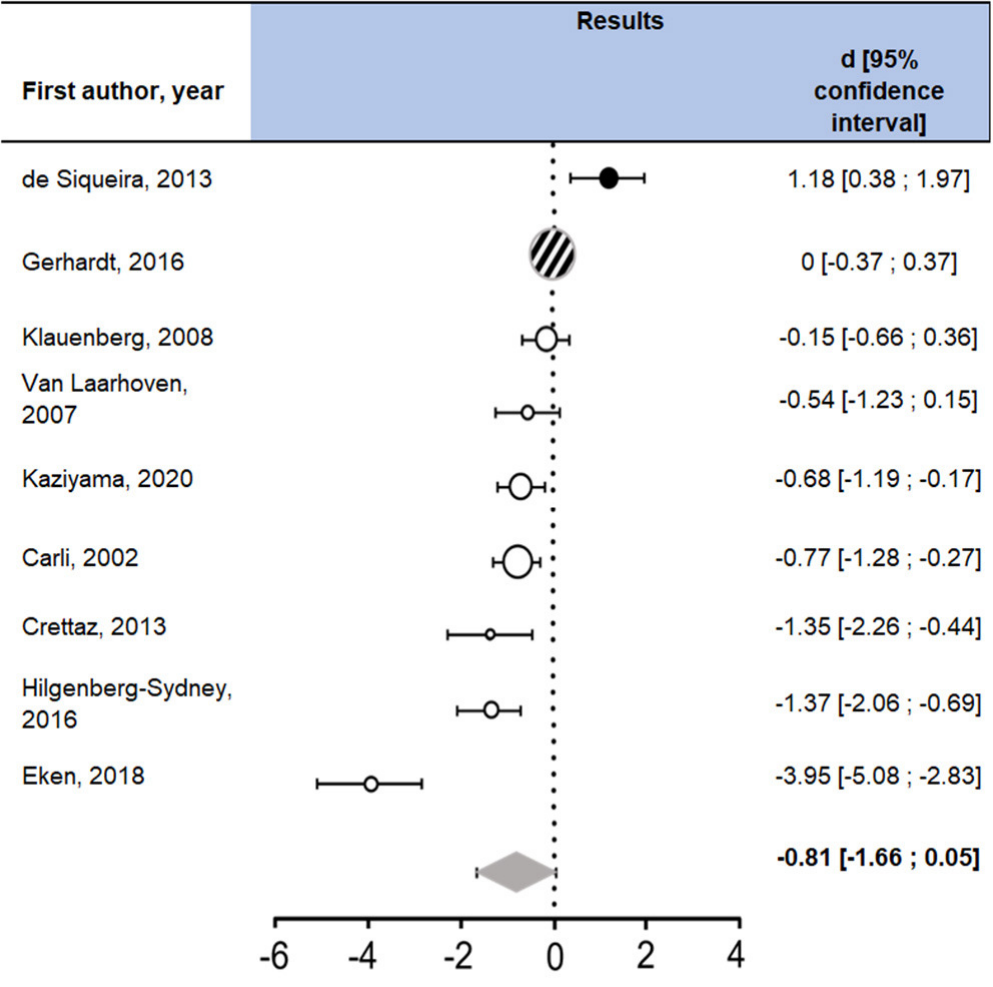
Studies included in the meta-analysis with measures of TPT. FM, participants with fibromyalgia; HC, healthy controls; SD, standard deviation. Question marks indicate non-reported data. A positive d (in black) can be interpreted as a hypoalgesia in participants with fibromyalgia compared to healthy controls, whereas a negative d (in white) means there is a hyperalgesia in participants with fibromyalgia compared to healthy controls. Gerhardt et al.'s study indicated a d equal to zero, represented by a striped circle. The summarized Cohen's d is represented by the gray diamond. Confidence intervals containing zero means the d is not statistically significant.

The nine studies included in the meta-analysis resulted in a non-significant D of −0.81, with a tendency toward hyperalgesia in fibromyalgia (Figure 3). Cochran's Q showed a significant heterogeneity [Q (df = 8) = 73.37, *p* < 0.0001]. The study with the extreme result (62) was of moderate quality (score of 79%). No notable difference was found between this study and the rest of the studies included in the meta-analysis.

The data suggest a trend toward hyperalgesia in fibromyalgia, though the heterogeneity of the results prevents a definitive conclusion.

## Discussion

This work is the first to systematically review findings from studies assessing sensitivity to noxious and non-noxious tactile stimuli in individuals with fibromyalgia. Findings were yielded from 19 studies with an overall high quality and the results of 12 of these studies were synthesized in a meta-analysis. Despite the heterogeneity of the results, the data suggest a trend toward hyperalgesia and no difference of sensitivity to non-noxious tactile stimuli in participants with fibromyalgia compared to healthy controls, at least outside of tender points, which contradicts the hypothesis of a general increase in responsiveness of the central nervous system to noxious and non-noxious stimulations in fibromyalgia (65, 66). In this section, the implications of these results and the substantial heterogeneity of the results of the studies included will be discussed, then the limitations of this review will be examined, and finally some perspectives will be proposed.

Several studies reported differences in cerebral activation evoked by noxious stimuli in individuals with fibromyalgia compared to controls. In a study in which participants received noxious stimuli of similar intensity, individuals with fibromyalgia showed higher activation in pain-related areas (operculo-insular regions, anterior cingulate cortex, basal ganglia, parietal cortex) and motor areas (motor cortices, supplementary motor area, cerebellum) compared to controls, and rated the stimulation as more painful (67). In studies in which subjective pain intensity was matched rather than the noxious stimuli (leading to lower stimuli applied in participants with fibromyalgia), individuals with fibromyalgia displayed higher activation of pain-related regions including the primary and secondary somatosensory cortices (68, 69), the anterior insula (67), the anterior cingulate cortex (69), and motor regions such as the supplementary motor area and the basal ganglia (67). They also showed less activation of the thalamus (69) and reduced functional connectivity in the pain inhibitory network [between the anterior cingulate cortex and the amygdala, the hippocampi, and the brainstem and between the thalamus and the orbitofrontal cortex (70)], compared to controls. Moreover, in an event-related potentials study, the amplitude of laser-evoked potentials N170 and P390 was higher and broader at central and frontocentral electrodes in participants with fibromyalgia compared to controls (71).

Differences in cerebral activation evoked by non-noxious stimuli were also observed between persons with fibromyalgia and healthy controls. Two studies using electroencephalography, one studying event-related potentials and the other oscillation frequencies, showed divergences between the cerebral responses of the two groups (72, 73). Montoya et al. (72) found that the same non-noxious pressure stimulation elicited lower event-related potentials in the primary somatosensory cortex in the fibromyalgia group while Fallon et al. (73) identified a suppression of beta oscillations in individuals with fibromyalgia in the primary and secondary somatosensory cortices, and the insula. However, the detection of the non-noxious stimulations was not measured in these studies. In a functional magnetic resonance imaging study, Cook et al. (74) assessed the detection of warmth in participants with fibromyalgia and healthy controls. They reported no differences between the groups at the behavioral level but found more activation in the anterior cingulate cortex, the insula, the prefrontal cortex, and the supplementary motor area during the stimulation in the fibromyalgia group. These results suggest an amplified cerebral response to noxious and non-noxious stimuli in fibromyalgia, which, for the latter, is not necessarily accompanied by a decreased perceptual threshold. Although it might appear contradictory, it is important to keep in mind that the detection of the stimulus is only one of many steps in the processing of somatosensory stimuli.

In the present study a trend toward hyperalgesia was detected. Contrary to our expectations, no hyperesthesia was observed; in fact, if anything, the trend for abnormal sensitivity to non-noxious stimulations was in the opposite direction, that is toward hypoesthesia. This is consistent with the generalized hypervigilance hypothesis, which claims that persons with fibromyalgia allocate more attention to aversive and noxious stimuli (75–77). It was born as an explanation for studies reporting heightened perception of various aversive stimuli [pressure (78, 79), thermal (22), auditory, and visual (80)] in individuals with fibromyalgia and could lead to amplified perceived pain intensity and frequency (81, 82).

However, studies assessing allocation of attention on aversive stimuli reported conflicting results. Gonzalez et al. (83) examined attentional allocation to aversive non-somatosensory stimulations in an emotional Stroop task and reported that participants with fibromyalgia displayed a tendency to allocate more attention (i.e., they were slower at reading the colors of the words) to negative words and words characterizing fibromyalgia symptoms, related to positive words, but also showed a significant attentional bias to neutral words (vs. positive words), in comparison to controls; the attentional bias was therefore not specific to aversive stimuli and was generalized to neutral stimuli. On the other hand, in a dual task requiring the detection of noxious tactile stimuli and innocuous visual stimuli, Peters et al. (84) reported that participants with fibromyalgia were not better at detecting the noxious tactile stimuli compared to healthy participants and concluded that no alterations in attention were observed in this group.

The lack of altered sensitivity to non-noxious stimulations could indicate that alterations of unimodal processing of somatosensory information are not generalized to both noxious and non-noxious stimuli in individuals with fibromyalgia. Thus, the sensorimotor impairments (3–5) and distortions of body representations (6–10) observed in this syndrome cannot be explained only by perturbations of unimodal processing of somatosensory information. An important aspect to keep in mind is that only detection thresholds were assessed in this study. Therefore, alterations in higher level processes, such as multimodal processing, could still be altered. Further research on the integration of somatosensory information among other sensory and motor information in fibromyalgia could shed a light on these mechanisms. Another phase of unimodal processing could also be altered. Auld et al. (85) proposed a dissociation between tactile registration (i.e., “the initial awareness of sensory information”), measured by TDT and TPT; and tactile perception, which involves “processing registered stimuli to create an internal representation with understanding of spatial, temporal, and modality-specific characteristics” and is assessed with higher-level tasks including identifying an object by touching it (i.e., stereognosis) or localizing a tactile stimuli with eyes closed. Given this dissociation, alterations of tactile perception in fibromyalgia cannot be dismissed and future research on unimodal somatosensory processing should focus on this aspect.

The tests of heterogeneity concluded on significant divergences between the results of the studies of the meta-analysis. Heterogeneity in the clinical characteristics of individuals with fibromyalgia is a possible cause. In the studies included, average pain intensity ranged from mild [22.7/100; (34)] to severe [9/10; (59)], which indicates variations of severity across participants. To compensate for this variability, the fibromyalgia group was split into subgroups in two studies (31, 34). In the literature, no consensus for dividing individuals with fibromyalgia into subgroups exists and various criteria are used (86–88). Moreover, the present results encompass studies published over several decades, with different diagnostic criteria.

Differences in medication could also introduce a bias. Participants with fibromyalgia were instructed to stop taking their medication prior to their participation in less than half of the studies of the review [47%; (27, 30, 32, 33, 57, 59, 61–63)] and of the meta-analysis [42%; (32, 57, 59, 61, 62)]. Medication commonly used to alleviate fibromyalgia symptoms have been shown to reduce sensitivity to somatosensory stimuli (89–91) and to have an effect on cerebral activation of pain processes (91). However, for ethical reasons and to prevent a recruitment bias, the interruption of medication is not always the solution. Finally, discrepancies in the duration of the participation could have influenced the results. Some studies involved only a short QST [e.g., van Laarhoven et al. (37)] while others consisted of a long QST accompanied by other measures [e.g., Gerhardt et al. (61)]. These divergences could lead to disparities in attention and fatigue, and impact the obtained measures (92, 93).

This study has several limitations. Only nine and seven studies were included in the meta-analysis TPT and TDT, respectively. This was partly due to our inability to retrieve all missing data to calculate Cohen's d for each group comparison.

Additionally, to reduce the variability of our results and facilitate their interpretation, measures on the distal upper limb area only were included in the meta-analysis. This further reduced the number of studies included and prevents from generalizing the results to all body parts. However, even though various sites are comprised in this area (i.e., finger, thenar, dorsum of hand, wrist, forearm) and pertains to various dermatomes (94, 95), Cohen's d normalizes these discrepancies. Moreover, none of these sites are tender points.

In conclusion, the systematic review and meta-analysis show no alterations of detection of tactile stimulations in individuals with fibromyalgia, compared to healthy controls, apart from a trend toward hyperalgesia. Considering the heterogeneity of the studies, future research need to investigate whether alterations in higher-level processes, such as multimodal integration of sensory information, are present and could contribute to sensorimotor deficits and anomalies of body representation in fibromyalgia. Indeed, some recent studies point toward deficits in visuoproprioceptive integration in this syndrome (96, 97).

## Data Availability Statement

The original contributions presented in the study are included in the article/Supplementary Material, further inquiries can be directed to the corresponding author/s.

## Author Contributions

All authors listed have made a substantial, direct, and intellectual contribution to the work and approved it for publication.

## Funding

TA was supported by a fellowship from the Centre interdisciplinaire de recherche en réadaptation et integration sociale (Cirris) and CM was supported by an Emeritus salary award from the Fonds de la recherche Québec-Santé (grant no: #251649).

## Conflict of Interest

The authors declare that the research was conducted in the absence of any commercial or financial relationships that could be construed as a potential conflict of interest.

## Publisher's Note

All claims expressed in this article are solely those of the authors and do not necessarily represent those of their affiliated organizations, or those of the publisher, the editors and the reviewers. Any product that may be evaluated in this article, or claim that may be made by its manufacturer, is not guaranteed or endorsed by the publisher.

## Abbreviations

FM: participants with fibromyalgia
HC: healthy controls
TDT: tactile detection threshold
TPT: tactile pain detection threshold.
